# Supplementary material for: The Midbrain Preisthmus: A Poorly Known Effect of the Isthmic Organizer
Source: Int J Mol Sci. 2023 Jun 5;24(11):9769. doi: 10.3390/ijms24119769 (PMC10253667; doi:10.3390/ijms24119769)
Supplement: Supplementary file 1 [file ijms-24-09769-s001.zip › ijms-2371599-supplementary.pdf]

Table S1. List of studied genes.

Signal	preisthmus	
Ascl1 /Mash1	E11.5-,E13,5+	
Astn2	P56 -E15-	
Bcan	radial glia	
Bhlhe22	m2alar	
C1ql2	PAGm2	
Cacna2d1	-	
Calb1	E18,5	
Calb2	E18+SubB	
Cbln2	E18	-
Cck	IF, PAG+ A8+	
Cdh13	PAGm2, CuF	
Chrna2		
Col6a1	PAG+	
Ctnnb1		
Dach1	SubB+	
Dlk1	IF, PAG, CnF	
Ebf1		
Ebf3	SubB+ CnF+	
Ecel1		
En1	IF, A8, ToIC+, CnF+	
En2	IF, A8, ToIC+. CnF	
Enc1		
EphA7		
Fgf15	alar E11, E13, E15, ToIC	
Fgf8		
Foxp1	CnF+ SubB	
Foxp2	CnF+ E18.5	
Foxp4	E11.5	
Fxyd6	P56, PAG, Cnf	
Gad1	m2 alar	
Gad2	m2 alar	
GATA2	basal	
GATA3	basal, CnF+	
Gfra1	VTA, IF, CnF+	
Id2	ICtor?	
Id4	SubB	
Irs4	E11.5-E18.5	
Irx1		
Irx2		
Irx3		
Irx4		
Irx5		
Irx6		
Kctd4	SubB, CnF, PAGm2	
Lhx1/Lim1	E13,5-P4	
Lhx2	E11.5-E18.5, SubB, PAGm2, Ictor	
Lhx5	E13,5-E18,5	
Lhx9	E11.5-E18.5	
Lmx1a	A8, floor, VTAm2	

Lrrtn1	lim m2/+r0-
Lypd1	SubB, CnF, PAGm2
Meis2	
Myt1	E13.5
Myt1l	E13..5-E18.5
Nell2	P56 Sag, CuF, PAGm2
Nhlh1	
Nhlh2	IF, PAGm2
Nkx2.2	SubB, Cnf, PAGm2
Nnat	PAGm2, CnF, SubB
Npy	PAGm2, CnF, SubB
Nrcam	E13.5 m2alar
Nrp1	PAGm2, CnF, SubB
Nts	Rru, SubB, CnF, PAGm2
Otp	basPAGm2
Otx1	
Otx2	full m2
Pax2	E11,5-13,5
Pax3	
Pax5	E11,5-P56; IF
Pax8	E11,5-P28; IF
Pbx1	IF, SubB
Peg10	
Penk	m2
Pitx1	VTA, SubB sin SubB full E11.5-E15.5
Plch1	
Plxna1	Tor-IC?
Pou3f2	Tor-IC?
Pou3f3	Tor-IC?
Pou4f1	Tor-IC? Mes5
Pou6f2	PAGm2bas, SubB, CnF
Reln	Tor-IC?
Rgs4	PAGm2bas
Rprm	PAGm2bas, SubB
Sall3	Rru, vzm2
Sema3f	
Sfrp1	vz, E11.5, E13.5
Sim1	
Six3	SubB, CnF
Slc6a3	IF, A8
Slx17a6	Rru, CnF, PAGm2alar
Slc17a8	DRm2
Slx32a1	SubB, CnF, PAGm2
Slit1	E11.5-E13.5
Sox14	m2alar
Sst	PAGm2bas, Rru, Tor-IC
Tac1	
Tal1	Rru, SubB, CnF
Tal2	PAGm2bas, SubB, CnF, PAGm2alar
Tcf7l2	

Trh	alar, basal
Wnt1	limit m2-rh0
Wnt8b	
Zbtb20	Drmes, m2alar E13.5
Zeb2	m2alar
Zfhx3	
Zfhx4	

Alar
